# 2D Ultrasonic Antenna System for Imaging in Liquid Sodium

**DOI:** 10.3390/s19194334

**Published:** 2019-10-08

**Authors:** Léonard Le Jeune, Raphaële Raillon, Gwénaël Toullelan, François Baqué, Laura Taupin

**Affiliations:** 1 French Alternative Energies and Nuclear Energy Commission—Laboratory for Integration of Systems and Technology CEA-LIST, 91191 Gif-sur-Yevtte Cedex, France; Raphaele.RAILLON@cea.fr (R.R.); gwenael.toullelan@cea.fr (G.T.); laura.taupin@cea.fr (L.T.); 2 French Alternative Energies and Nuclear Energy Commission—Division of Nuclear Energy CEA-DEN, 13108 Saint-Paul-lez-Durance Cedex, France; francois.baque@cea.fr

**Keywords:** SFR, in-service inspection, imaging, ultrasonic transducer, NDT (Non Destructive Testing), NDE (Non Destructive Evaluation)

## Abstract

Ultrasonic techniques are developed at CEA (French Alternative Energies and Nuclear Energy Commission) for in-service inspection of sodium-cooled reactors (SFRs). Among them, an ultrasound imaging system made up of two orthogonal antennas and originally based on an underwater imaging system is studied for long-distance vision in the liquid sodium of the reactor’s primary circuit. After a description of the imaging principle of this system, some results of a simulation study performed with the software CIVA in order to optimize the antenna parameters are presented. Then, experimental measurements carried out in a water tank illustrate the system capabilities. Finally, the limitations of the imaging performances and the ongoing search of solutions to address them are discussed.

## 1. Introduction

Since France considered in 2008 that the sodium-cooled reactor (SFR) concept was the most mature for Generation IV nuclear reactors, an extensive R&D programme was launched. In-service inspection was identified as a difficult task to be performed (as the sodium coolant is opaque, hot, highly chemically reactive, and difficult to drain). Ultrasonic techniques have been extensively studied as they are well adapted to Non Destructive Evaluation (NDE) and telemetry measurement in this harsh environment.

Thus, development of ultrasonic transducers to be immersed in sodium at about 200 °C in the reactor block (inspection is performed at shutdown conditions) led to a first phase of specification consolidation; then, a prequalification process involving increasingly more realistic experiments using acoustic techniques and simulations was performed with the patented CIVA code [1].

Associated applications for inspection deal with telemetry, vision, and volumetric control for SFR reactor block systems, structures, and components and for the power-conversion system.

Some techniques deal with short-distance imaging such as the EMAT (Electromagnetic-Acoustic Transducer) of CEA [2] and TUCSS (Ultrasonic transducer for Non Destructive Testing—NDT—of structures immersed in liquid sodium at 200 °C) of FRAMATOME [3] that are being developed for NDE, whereas other, such as the orthogonal imaging concept based on an underwater imaging system [4] and developed in the 2000s [5], deals with long-distance vision and the exploration of a large area without moving the probe. The orthogonal imaging system allows for scanning a much larger area than that scanned with the same number of elements arranged in a matrix pattern, and thanks to the large antenna’s aperture in the focusing plane, remote targets are likely to be imaged. This concept has been taken up recently in order to reexplore its capabilities using actual simulation tools and imaging methods. The first simulated results and experimental measurements performed in water highlight the potential of the system in which manufacturing for sodium trials is ongoing at Toshiba Corporation while imaging algorithms are specifically adapted or developed for its applications.

## 2. Imaging Principle of the 2-Antenna System

### 2.1. Description of the 2-Antenna System

**Figure 1:**
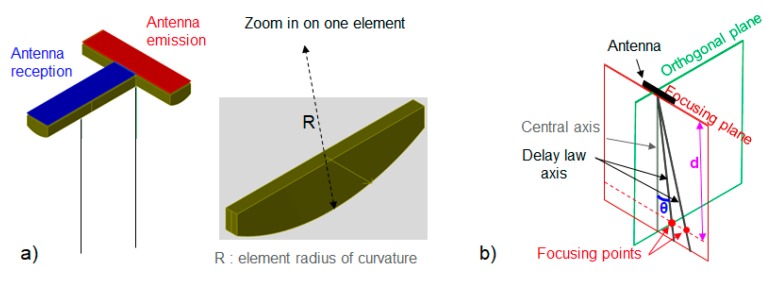
Diagram of the antennas’ arrangement and definition of the main terms used in the article: (**a**) Diagram of the two antennas’ arrangement and of the orthogonal section of an element and (**b**) definition of the key terms for one antenna. The central axis is perpendicular to the probe surface at its centre. The delay law axis represents the direction of the deflected beam when delay law is applied. When a delay law is applied, the beam is focused at the focusing point placed at a distance *d* as defined in the figure and or deflected by an angle θ in the focusing plane of the antenna. The orthogonal plane is perpendicular to the focusing plane.

The 2-antenna system is made up of two identical linear-phased arrays disposed in a “T” arrangement (Figure 1a) which allows three-dimensional imaging. One antenna operates in emission (E; the red one) and the other in reception (R; the blue one). The surface of each element of the arrays is planar in the focusing plane and convex with a radius of curvature R in the orthogonal plane of the antenna (Figure 1a,b). The values of the antenna parameters are provided later.

### 2.2. Imaging Using Electronic Scanning 

**Figure 2:**
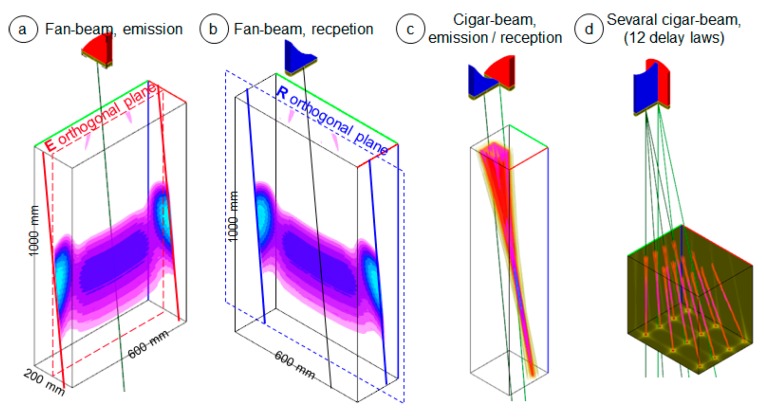
Simulated beam results: Illustration of the electronic scanning principle by visualization of the antenna beams computed with CIVA in a 3D zone of which the dimensions are given on the figure. (**a**) Fan-beam radiated by the emission antenna. (**b**) Fan-beam radiated by the reception antenna. (**c**) Emission/reception (E/R) cigar-beam. (**d**) Illustration of the twelve cigar-beams obtained for twelve delay laws (the beams are displayed together in the same image, but in reality, the twelve delay laws are applied successively to scan the space).

A delay law is applied to each antenna in order to focus its beam at a chosen focusing point in its focusing plane (Figure 2a,b) and, thus, to obtain a very narrow beam in this plane, whereas, due to the convex surface of the elements, the beam is very divergent in its orthogonal plane. Due to its shape, the beam of each antenna is called a “fan-beam” [5].

The intersection of the two fan-beams creates the useful part of the beam in the imaging process, named “cigar-beam” because of its shape, and is oriented along an axis located between the E delay law axis and the R delay law axis (Figure 2c). Thus, by applying successive delay laws in order to move the “cigar-beam”, it is possible to scan and image a 3D zone (Figure 2d).

### 2.3. Imaging Using a Full Matrix Capture (FMC) and the Total Focusing Method (TFM)

**Figure 3:**
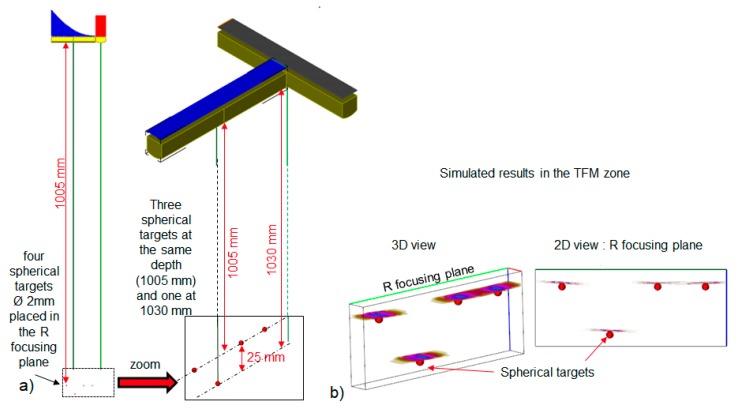
Simulated Full Matrix Capture (FMC)/Total Focusing Method (TFM) results: (**a**) Position of the spherical targets in front of the two antennas. The four spheres are in the R focusing plane (left); three of them are at a 1005-mm depth and one is at a 1030-mm depth (right). (**b**) Three-dimensional and 2D TFM images of the spherical four targets computed in a 3D zone placed around the targets as shown in Figure 3a.

To image the 3D area in front of the antennas, it is also possible to use the total focusing method after an FMC acquisition [6]. In this case, the FMC is performed by firing each element of the transmitting probe (E) and by receiving on every element of the receiving one (R). This imaging method is available in CIVA, and a simulated example of TFM applied for spherical targets is displayed Figure 3.

### 2.4. Main Advantages and Drawbacks of the Imaging System

As said in the introduction, the main advantage of the 2-antenna system is the large aperture of each antenna, much larger than that obtained with the same number of elements arranged in a matrix pattern, allowing the improvement of the spatial resolution (see, for example, numerical values of the spatial resolution in Section 3.2). The main drawback comes from the way the cigar-beam is formed from the two fan-beams. Indeed, as the combination of the two fan-beams leads to a deflected cigar-beam of which the orientation depends on the position of the focusing point in the field of vision, the system is suitable only for targets generating specular echoes that come back to the receiver regardless of their orientation and position in the field of vision (as spherical targets or targets with rounded parts) and for targets, as the large, rough, curved surfaces of a reactor vessel, that diffract the field in all directions (that is the main application presented in Reference [5]). A distortion could be observed for targets generating specular echoes that do not come back to the receiver, but some targets could also be not detected at all if the specular echoes completely miss the receiver. For targets for which low diffraction echoes are involved in the imaging process, a small signal-to-noise ratio (SNR) is obtained. These considerations on the orientation of the cigar-beam used for the scanning imaging are also valid for the elementary beams impacting the target in the FMC/TFM imaging. Thus, the elementary echoes of the FMC might be invisible and lost in the noise, with the total target echo emerging from the noise only after the TFM computation.

## 3. Simulation Study for Antenna Parameter Optimization

Whatever the imaging method applied (scanning or TFM), the same requirements must be fulfilled regarding the radiated beam of the system to ensure an effective imaging in the case of the targeted applications (mainly far-field imaging of lost objects and detection of structure displacement). These requirements lead to the same optimized values of the antenna system parameters for both imaging methods.

### 3.1. Required Beam Features and Associated Influencing Parameters

The first requirements deal with the dimensions of the field of vision. In the orthogonal plane of each antenna, an almost constant beam’s amplitude is required at each depth. In the direction of the focusing plane of each antenna, the potential grating lobes should be avoided. For depth, the amplitude should not decrease too quickly as long-distance imaging is contemplated. Other requirements concern the focal spot of the cigar-beam (axial and longitudinal resolutions). 

The antenna parameters impacting these beam features are the classical phased array parameters such as the element size in the focusing plane, the gap between two adjacent elements, and the number of elements (these three parameters define the probe total aperture in the focusing plane) and also the antenna element’s curvature and aperture in the orthogonal plane.

The effects of all these parameters in various combinations were evaluated and optimized with the CIVA software in relation to both the signal centre frequency fixed between 1 MHz and 2 MHz according to previous propagation studies in liquid sodium and to the bandwidth imposed by manufacturing constraints (about 30%). Some results of the CIVA study are presented in the next paragraph.

### 3.2. Example of the Effects of Some Influencing Parameters on the 2 Antenna Beams 

**Figure 4:**
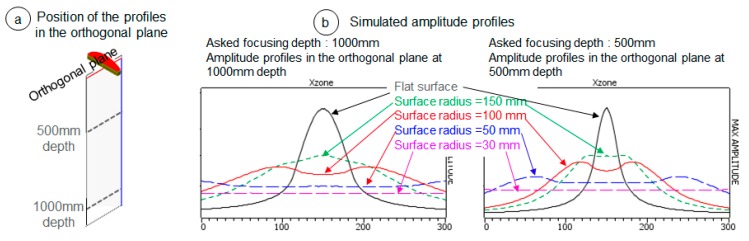
Simulated beam results: Effect of the variation of the radius of curvature of the elements in the orthogonal plane on the radiated beam at two depths. (**a**) Position of the profiles (dash lines) where the beam was computed. (**b**) Simulated amplitude of the beams along the 2 profiles obtained for different radii of curvature of the elements. The fixed parameters used for the computation were 128 elements for each antenna, with element’s lengths of 1.8 mm in the focusing plane and of 35 mm in the orthogonal plane, a gap of 0.2 mm between adjacent elements, a centre frequency of 1.6 MHz, and a bandwidth of 30%.

The element’s surface curvature and length in the orthogonal plane affect the beam divergence. For example, we present the study on the effect of the surface curvature. The simulated beams of one antenna (Figure 4) show that, when the element surface becomes more and more curved, from a flat surface to a curved surface with R = 30 mm, the beam divergence increases in the orthogonal plane, leading to a decrease in the radiated amplitude at a given depth. Thus, the chosen curvature was a trade-off between the divergence—required to ensure at each depth a constant amplitude in a large angular aperture—and the sensitivity. 

**Figure 5:**
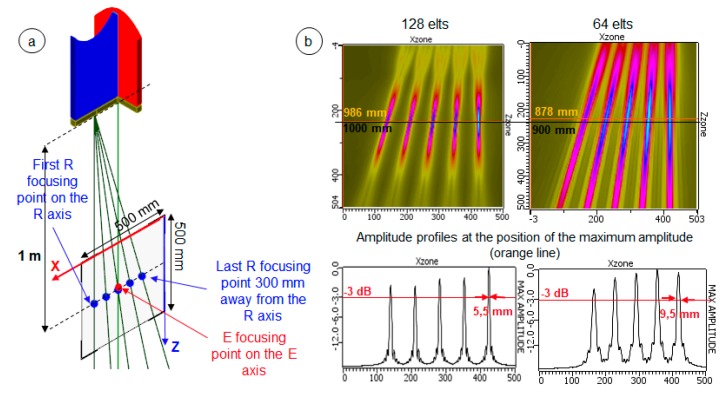
Simulated beam results: Effect of the variation of the antenna’s aperture in the focusing plane on the cigar-beam spatial resolution at a given depth. (**a**) Position of the 2D beam computation area. (**b**) Simulated maximum amplitude of the beams obtained in this area for antennas with 64 elements (i.e., for an aperture of 127.8 mm) and with 128 elements (i.e., for an aperture of 255.8 mm). The fixed parameters used for the computation were element’s lengths of 1.8 mm in the focusing plane and of 35 mm in the orthogonal plane, a gap of 0.2 mm between adjacent elements, a surface radius of curvature of 30 mm, a centre frequency of 1.6 MHz, and a bandwidth of 30%.

The antenna aperture affects the spatial resolution as illustrated in Figure 5. As expected, the larger the aperture, the better the spatial resolution. To increase the antenna aperture, we can increase the element length, the pitch, or the number of elements. The element’s length and the gap (i.e., the pitch) are imposed at the operating centre frequency as their increase leads to the presence of grating lobes. Then, we choose to increase the number of elements knowing that it will be limited by the maximum number of elements that can be operated and by manufacturing constraints. We hoped to build 128 elements antennas, but we know now that the number of elements will be comprised between 64 and 75.

For example, with 128 elements (i.e., with an aperture of about 256 mm), when a delay law is applied to focus the emitted beam of one antenna at a point placed at a 1000-mm depth on its central axis, the focal spot dimension in its focusing plane is 5.5 mm. While, with 64 elements (i.e., with an aperture of about 128 mm) and the same point of focalization, the focal spot dimension increases to 9.5 mm (see Figure 5, bottom right).

Eventually, taking into account the simulation study performed with CIVA to optimize the antenna parameters for 3D imaging in liquid sodium at 200 °C leads to the following set of values for the antenna parameters. The number of elements is 128, the element length in the focusing plane is 1.8 mm, the gap between two adjacent elements is 0.2 mm, the element dimension in the orthogonal plane is 20 mm, the radius of the convex surface in the orthogonal plane is 30 mm, the centre frequency is 2 MHz, and the bandwidth of the excitation signal is about 30% with respect to the centre frequency. 

## 4. Experimental FMC/TFM Images Performed in Water and Associated Simulations with CIVA

**Figure 6:**
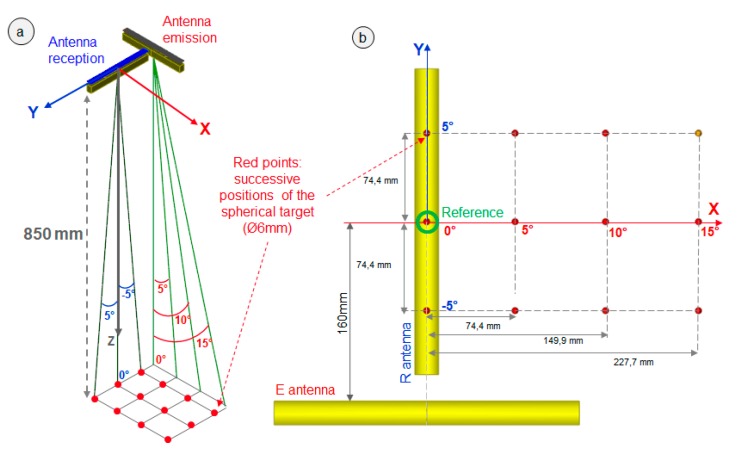
Diagram of the experimental trial configuration: (**a**) 3D diagram of the two-antenna system and successive positions of the spherical targets at a 850-mm depth (red points): the spherical target’s FMC were measured one after the other for each of the twelve positions of the sphere. (**b**) Top view of the same configuration. The sphere located at X = 0° and Y = 0° (surrounded by a green circle) is used as a reference for the amplitudes (see in the text).

A two-antenna prototype was designed in 1999 by CEA and manufactured by IMASONIC French company to perform tests in a water tank using large curves and rough surfaces [5]. The number of elements of each antenna is 128, the element length in the focusing plane is 1.8 mm, the gap between two adjacent elements is 0.2 mm, the element dimension in the orthogonal plane is 20 mm, the radius of the convex surface in the orthogonal plane is 35 mm, the centre frequency is 1 MHz, and the bandwidth of the excitation signal is 50%. The relative positions of the two antennas are given in Figure 6.

Various experimental FMC and TFM images of spherical and planar targets were performed in order to assess the antennas’ capabilities in terms of sensitivity and spatial resolution. The associated FMC and TFM were also computed with CIVA (the model used for the echoes computation of the spherical targets presented below was the SOV (Separation of Variable) model). For example, some FMC acquisitions of the echoes of a spherical target (diameter Ø = 6 mm) located at a 850-mm depth were carried out for 12 different positions of the target in the antennas field of vision (Figure 6). 

**Figure 7:**
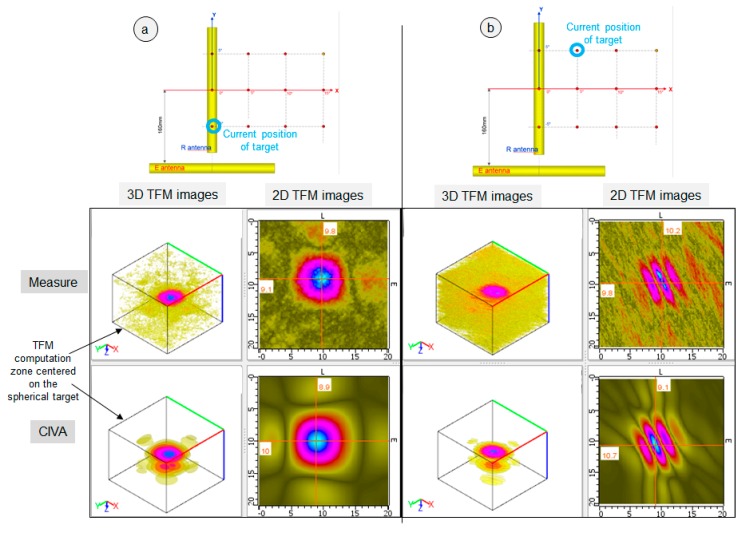
Measured and simulated FMC/TFM results: (**a**) The spherical target is at the position surrounded with a blue circle in the 2D diagram of the experimental trial configuration (top of the figure). (**b**) The spherical target (Ø: 6 mm) is at the position surrounded with a blue circle in the 2D diagram. For each position of the target, the measured and CIVA simulated 3D and 2D TFM images are displayed. The 3D TFM image was computed in a 3D zone centered on the spherical target and represented on the figure. The 2D TFM image is in the XY plane and was extracted from the 3D image at the position of the maximum amplitude.

**Figure 8:**
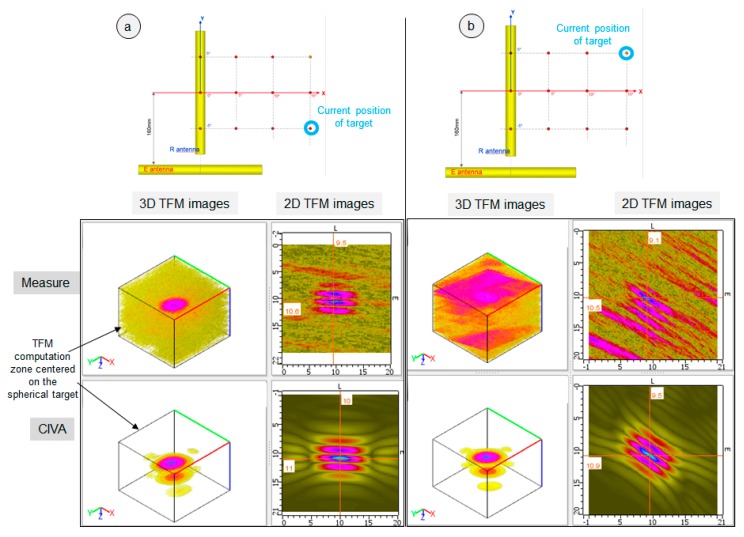
Measured and simulated FMC/TFM results for targets placed far from the antenna: (**a**) The spherical target is at the position surrounded with a blue circle in the 2D diagram of the experimental trial configuration (top of the figure). (**b**) The spherical target (Ø: 6 mm) is at the position surrounded with a blue circle in the 2D diagram. For each position of the target, the measured and CIVA simulated 3D and 2D TFM images are displayed. The 3D TFM image was computed in a 3D zone centered on the spherical target and represented on the figure. The 2D TFM image is in the XY plane and was extracted from the 3D image at the position of the maximum amplitude.

The TFM measured images obtained for four positions of the sphere are presented Figure 7 and Figure 8. They show the good capability of system imaging as, even for the target far from the R antenna axis, the TFM image’s quality is good in terms of SNR and resolution in the three cases of Figure 7a,b and Figure 8a. It is only when the sphere is far from both the E and R antenna axes (Figure 8b, where the sphere is distant 235 mm from the E antenna and 227 mm from the R antenna) that the SNR becomes too low to allow a good experimental image of the sphere. The SNR values measured on the experimental TFM are 9.5 dB for Figure 7a, 8 dB for Figure 7b, 8 dB for Figure 8a, and 2 dB for Figure 8b. It should be noted that, to calculate these SNRs, both attenuation and grating lobes were included in the noise measurement.

The four results also show a good qualitative agreement between CIVA and the measures regarding the evolution of the target image with the position of the sphere in the field of vision. A good quantitative agreement was also obtained for the maximum amplitude of the TFM images: the discrepancy between CIVA and the twelve measures was less than 3 dB (the reference for the amplitude is the TFM amplitude obtained for the sphere located at X = 0° and Y = 0°, which is surrounded by a green circle in Figure 6). However, the model used for the FMC simulation in CIVA does not predict the noise observed on the experimental image as it is not taken into account for the computation.

## 5. Discussion for Future Work: Improvement of the Images’ Signal-to-Noise Ratio and their Computation Time 

It has been experimentally shown that the TFM applied to an FMC acquisition performed with the two-antenna system allows to image spherical targets at large distances in water. However, two main drawbacks have been identified. The first one is the low Signal-to-Noise Ratio (SNR), especially for far-reaching targets. The second one is the high computation time required to obtain the images, which is due to the large size of the field of vision (several hundred centimetres) and the large number of elements of the antennas. In order to optimize the SNR and the computation time, several leads are being investigated.

### 5.1. Signal-to-Noise Ratio Improvement

During full matrix capture, each probe’s element of the antenna operating in emission is fired individually and emits a spherical wave that propagates in the medium and gives rise to an elementary echo when meeting a target. This emission process, while it allows to insonify a large volume, transmits a limited acoustic power as only one element is used. In order to increase the acoustic power sent into the medium, the Plane Wave Imaging (PWI) method [7] can be used. The principle of this method is to emit plane wave fronts generated by all the elements of the probe at different angles instead of the spherical elementary wave front emitted by one element. As all the probe’s elements are excited together, the acoustic power sent in the medium is much higher. Then, for the FMC, the backscattered signals are recorded on every element of the probe and the signals are coherently summed up to obtain an image similar to the one obtained by TFM. The sole difference when applying the TFM is the computation of the incident wave’s time of flight (spherical for the FMC and plane for PWI). In order to take into account cylindrical wave fronts emitted by the element curved surface in the orthogonal plane, some modifications were added to the “classical” PWI method that deals with emitted planar wave fronts. Another possibility to improve the SNR is to use encoded transmissions like Hadamard spatial codes [8]. With this method, all the probe’s elements are excited simultaneously, but for every transmission, N electric signals applied to the N elements are multiplied by a combination of a +1/−1 coefficient. A coded matrix is thus formed. The equivalent impulse response matrix (i.e., FMC) can be obtained with a decoding operation. The Hadamard coded excitation increases the SNR by an $\sqrt{N}$ factor compared to a conventional FMC. 

**Figure 9:**
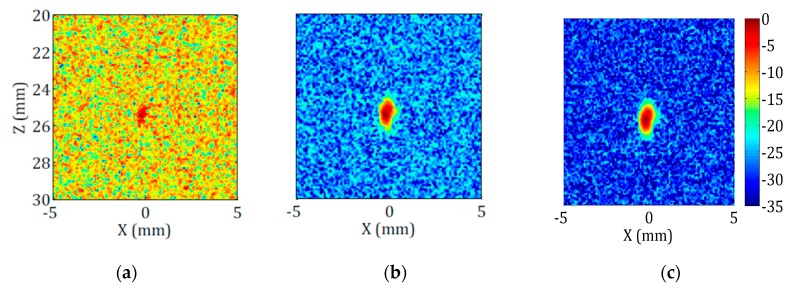
Comparison between (**a**) TFM, (**b**) Hadamard coding, and (**c**) Plane Wave Imaging (PWI) images obtained in a High-Density Polyethylene (HDPE) component for a 64-element, 5-MHz probe. The case is of a side-drilled hole (diameter of 2 mm) at a 25-mm depth. The amplification gain and voltage excitation are the same for the three acquisitions process. Attenuation of HDPE in the bandwidth is comprised between 0.4 and 1.4 dB/mm. Signal-to-Noise Ratios (SNR) are respectively 9 dB, 15 dB, and 18 dB [8].

The improvements resulting from the application of these two methods (PWI and Hadamard) are not illustrated for our configuration of interest because it requires new experimental trials, where the FMC will be replaced by a PWI or Hadamard acquisitions. These acquisitions are not completed at the moment. Thus, instead of illustrating for telemetry applications (long-distance case), we illustrate the methods for a highly attenuating medium (short-distance case). In each case, a high-level noise is induced by attenuation (by the propagation distance or the high attenuation coefficient) and the same methods can be applied to reduce it. Figure 9 shows the images obtained with three methods (TFM, Hadamard, and PWI) for a side-drilled hole (diameter of 2 mm) at a 25-mm depth in a strongly attenuating High-Density Polyethylene (HDPE) component with a 5-MHz, 64-element probe. The noticeable increase in SNR with the Hadamard coding and PWI demonstrates the benefit that can be expected from these methods.

### 5.2. Computation Time Improvement

The other axis for improvement is the computation time of the images. Three options are currently under investigation.

The first option is to use massive GPU (Graphic Processing Unit) parallelization. As the TFM, Hadamard, or PWI algorithms consist in a large number of operations (summations of amplitudes at a given time of flights for shot and receiving elements), they can be quite parallelized on a CPU (Central Processing Unit) or a GPU. In order to avoid poor scaling effects due to concurrent memory accesses, parallelization has been done on pixels and very good improvements in computation time have been obtained on both CPU and GPU architectures [9].

**Figure 10:**
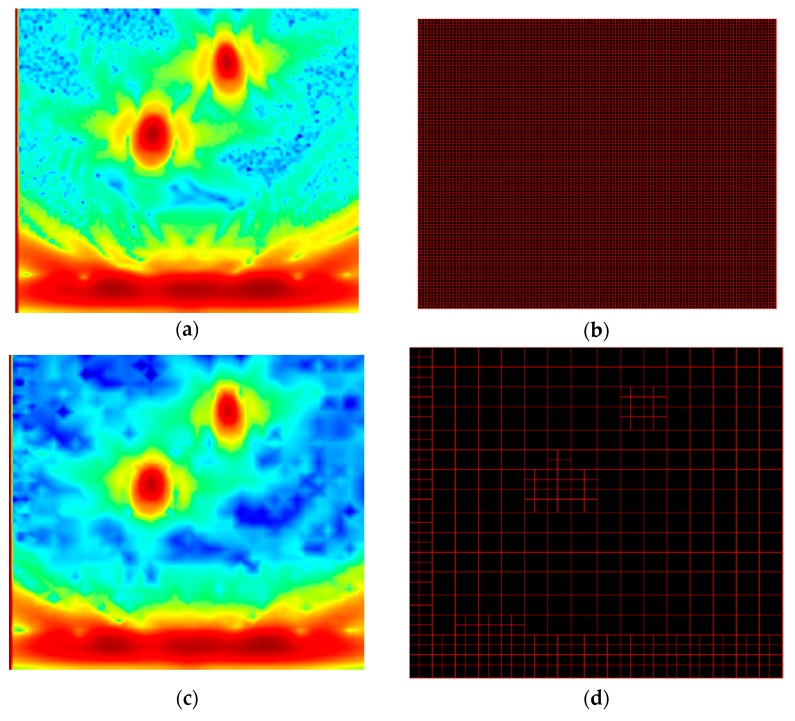
Adaptive grid: (**a**) TFM image obtained using (**b**) a regular and very fine grid and (**c**) TFM image obtained using (**d**) an adaptive grid. The number of pixels in Figure 10d represents 16% of the total number of points in Figure 10b.

The second option, which can be used with any method and with parallelization, is to develop an “adaptive” grid in the imaged area. Here, the principle is to start the imaging process with a loose grid, then to detect the high-amplitude regions, and to iteratively refine the grid around those regions [10]. In our configuration that is purely three-dimensional, a 3D grid is needed. Work is ongoing to develop it, and at the moment, we can illustrate it only with a 2D configuration. Figure 10 shows 2D TFM images obtained on a set of simulated data for two side-drilled holes in a steel specimen imaged with 64 elements and a 2-MHz probe. It can be noted that, in this case, the same image quality is achieved by the adaptive grid with only 16% of the number of pixels used in the original case (area of 45 × 40 mm with a regular step of 0.4 mm).

**Figure 11:**
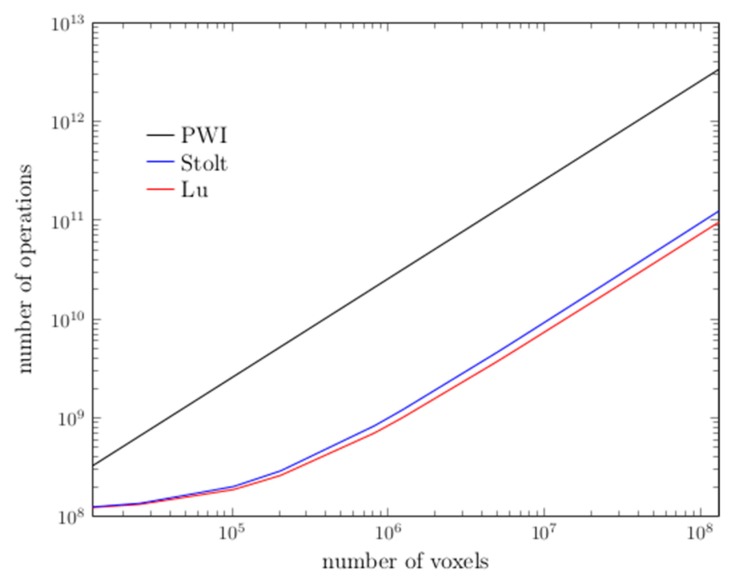
Algorithmic complexities: comparison between frequency domain (f-k) methods (Stolt and Lu) and PWI [11].

The last option is to use frequency domain (f-k) methods. These methods are well known to greatly reduce computation times. In particular, the methods developed by Stolt and Lu has been adapted to NDE cases and to plane wave imaging [11]. It has been shown that f-k methods greatly reduce the algorithm complexity (number of operations) compared to the classical PWI (cf. Figure 11) and, thus, the computation time. The result presented Figure 11 was obtained in pulse-echo mode, and the work to extend it to our antenna system, with separate transmission and reception, is ongoing.

Furthermore, it is important to note that our configuration presents two advantages regarding the computation time of the images that strongly depends on the time required to compute time of flight between the elements and the points of the imaged area. The first one is the absence of interface between the elements of the antennas and the inspected area, and the second one is that the propagation occurs in a fluid medium (case of the sodium at inspection temperature), where only longitudinal waves exist. Indeed, the time-of-flight computation requires knowledge of the wave path between the transmitting element and the focusing point. This path is much easier and thus faster to compute when there is no interface as there is no refraction of the waves. Imaging the large area of the antenna field of vision is made possible thanks to these advantages combined with the implementation of the methods to decrease the image’s computation time.

## 6. Imaging Acoustic Sensor for Sodium Conditions

This 2-antenna acoustic system is devoted to sodium viewing with the following applications: global imaging of the large primary circuit (some meters dimension), hypothetical large disorder detection (structure buckling), and potential lost-parts identification. The present paper exhibits the capabilities of such an enhanced imaging sensor which has been optimized with the CIVA software: experimental measurements were carried out in a water tank, and results show a good qualitative agreement between CIVA simulated results and the measures regarding spherical and planar target images. Having in hand this original concept of a 2-antenna acoustic system and the simulation CIVA code validated on commissioning water test results, it has been possible to design a first sodium prototype. Made of 64 curved elements for each antenna, it is being manufactured by Toshiba Corporation, which found and implemented specific technological solutions for bonding of the piezo material and backing material, front plate material, and array processing of the probe.

Commissioning tests of this prototype will be first performed in water in Japan by the end of 2019; then sodium tests are scheduled in France in 2020. Dealing with abovementioned applications for in-sodium vision, different targets will be used with different shapes: spheres for global viewing capacities of the antenna, flat surface with small basins to evaluate the antenna’s performances for surface metrology application, and nuts for the lost-parts detection performances.

## 7. Conclusions

We presented a technique for long-distance vision in liquid sodium that enables imaging of large areas using a system of two orthogonal linear phased arrays (antennas) and the total focusing method. The use of CIVA to compute the antenna-radiated beams allows to easily understand and illustrate the effect of all the antenna parameters (frequency, aperture, pitch, etc.). CIVA also enables to study many combinations of parameters that was not possible in the first studies of this system and to propose an optimized set of values regarding the targeted application of long-distance viewing. We presented some examples of this simulated parametric study. The comparison of CIVA simulations with experimental results shows a good agreement aside from the noise that is not taken into consideration because CIVA does not compute it. The FMC/TFM imaging method was used to image the antennas’ field of vision when, in the first study, the image was performed thanks to successive focalization at the different points of the field. This imaging method is very efficient as illustrated by the experimental results obtained in water, but as we deal with long-distance imaging and large imaging areas, small SNR and computation time appeared as limitations for this method. We proposed several leads to improve both of them that will be soon experimentally evaluated in water.
